# Retinal Sensitivity Correlates With the Superficial Vessel Density and Inner Layer Thickness in Diabetic Retinopathy

**DOI:** 10.1167/iovs.62.14.28

**Published:** 2021-11-30

**Authors:** Azzeddine Mokrane, Abir Zureik, Sophie Bonnin, Ali Erginay, Carlo Lavia, Alain Gaudric, Ramin Tadayoni, Aude Couturier

**Affiliations:** 1 Université de Paris, Ophthalmology Department, AP-HP, Hôpital Lariboisière, F-75010, Paris, France

**Keywords:** diabetic retinopathy (DR), capillary density, microperimetry, optical coherence tomography-angiography (OCT-A), retinal sensitivity

## Abstract

**Purpose:**

The purpose of this paper was to present our study on the relationship between the parafoveal sensitivity measured using microperimetry and the vessel density (VD) assessed by optical coherence tomography-angiography (OCT-A) in eyes with diabetic retinopathy (DR).

**Methods:**

The observational case series was conducted in a tertiary ophthalmology center. Eyes with DR and without macular edema were consecutively included. All eyes underwent microperimetry and OCT-A. The correlation between the regional retinal sensitivity and the corresponding local capillary changes and structural alterations seen on OCT-A was assessed in each retinal quadrant.

**Results:**

Thirty-seven eyes of 21 patients were included. The mean retinal sensitivity was 28.7 ± 2 decibel (dB). The mean parafoveal VD was 43.2 ± 4.2% in the superficial capillary plexus (SCP) and 48.1 ± 3.3% in the deep capillary complex (DCC). In the multivariate linear regression model, the mean retinal sensitivity was positively correlated with the VD in the SCP in the parafoveal ring (*P* = 0.01) and with the inner nuclear layer (INL) thickness (*P* = 0.01). The qualitative analysis of each quadrant showed the presence of areas of capillary dropout with a normal sensitivity. Conversely, all areas of decreased sensitivity (<25 dB) were associated with a decreased VD in the SCP and the DCC.

**Conclusions:**

The parafoveal sensitivity positively correlated with the VD in the SCP in DR eyes. Areas with a low retinal sensitivity were always co-located with a loss of capillaries in the SCP and the DCC despite preserved outer retinal layers.

Although capillary dropout is a hallmark of diabetic maculopathy, its role in visual function impairment is poorly understood. It is now well known that diabetic retinopathy (DR) results from several factors, including glial and neuronal cell dysfunction, inflammatory reactions, blood retinal barrier breakdown, and capillary dropout.^1^ Several studies using fluorescein angiography (FA) have attempted to correlate diabetic macular ischemia and visual acuity (VA) with various results, probably due to the inability of FA to show the deep capillary complex (DCC) and to the complexity of the factors involved in vision.^2^^,^^3^

The advent of optical coherence tomography angiography (OCT-A) has allowed for the first time analyzing both the superficial capillary plexus (SCP) and the DCC and calculating the vessel density (VD) and capillary nonperfusion areas in each plexus.^4^^–^^7^ OCT-A has been shown to have a high sensitivity for the detection of vascular changes in DR,^4^^,^^8^ and has revealed the early DCC impairment, which was undetectable by FA, in patients with diabetes with no or early stages of DR,^9^ or with diabetic macular edema (DME).^10^^–^^12^ Recently, the relationship between the DCC impairment and the VA loss has been highlighted in young patients with type 1 diabetes with severe DR.^13^ Other studies have assessed the relationship between visual function and anatomic parameters in patients with no DR or early DR.^14^^–^^16^ However, the relationship between the capillary closure and the visual function yet remains largely unknown in eyes with other DR grades, and several OCT-A studies have only reported a weak correlation between the VD and the VA in diabetic eyes.^10^^,^^17^^,^^18^

The retinal sensitivity measured using microperimetry provides a more complete and accurate evaluation of the macular function compared to the VA and may be an early marker of visual function loss in diabetic eyes, because it has been shown to be significantly reduced in patients with diabetes without DR.^19^^,^^20^ Recent studies have shown a correlation between the VD and the retinal sensitivity,^21^^,^^22^ but they have not specifically investigated the local relationship and consistency between nonperfusion areas and areas of decreased retinal sensitivity.

The aim of this study was thus to investigate the relationship between the parafoveal sensitivity measured using microperimetry and the macular VD and the presence of nonperfusion areas assessed using OCT-A in diabetic eyes and to assess the local changes in capillaries within areas of decreased retinal sensitivity in each retinal quadrant.

## Methods

### Participants

This was an observational case series conducted in a tertiary ophthalmology center (Lariboisière Hospital, University of Paris, Paris, France). Eyes of patients with diabetes, with or without DR, and without DME were consecutively included over a 4-month period (December 2017–March 2018). This study was approved by the Ethics Committee of the French Society of Ophthalmology (institutional review board [IRB] 00008855), and a written informed consent was obtained from all patients for the review of their records.

Inclusion criteria were patients ≥18 years with type 1 or type 2 diabetes, with or without DR diagnosed on fundus imaging. Both eyes of the patients were included if eligible.

Exclusion criteria were the presence of DME, defined as a central subfield thickening of at least 315 µm on spectral-domain optical coherence tomography (SD-OCT), corresponding to the normal value +2 standard deviations: 277 + (2 × 19) µm,^17^ with intra- or subretinal fluid; the presence of glaucoma; the presence of any other retinal disorder (including high myopia); a history of focal macular laser, recent cataract surgery (<4 months) or pars plana vitrectomy; a poor quality of images due to media opacity, such as vitreous hemorrhage or cataract. Poor quality OCT-A images were defined by a signal strength index (SSI) <60/100.

Data collected were demographics (age, gender, diabetes duration, hemoglobin A1c [HbA1c] level, and previous DR treatments) at the inclusion visit and current ophthalmologic examination findings, including the best-corrected visual acuity (BCVA [Snellen letter chart]), intraocular pressure, slit-lamp examination, and dilated fundus biomicroscopy.

Color fundus photographs and OCT-A images were obtained using the ultrawide field Optos imaging system (Optos PLC, Scotland, UK) and the Angiovue (RTVue XR; Optovue, Inc., Fremont, CA, USA), respectively.

The DR severity score (DRSS) based on the **Early Treatment Diabetic Retinopathy** Study (ETDRS) DR grading scale simplified by the American Academy of Ophthalmology (AAO) was used to classify DR as mild, moderate, or severe nonproliferative DR (NPDR) or proliferative DR.^23^

### Microperimetry

The retinal sensitivity was measured using the MAIA Microperimeter (Centervue, Padova, Italy) in all eyes. Microperimetry was performed in a darkened room under monocular testing conditions by occluding the non-tested eye, according to the manufacturer's instructions. The MAIA Microperimeter allows measuring the retinal sensitivity within a range from 0 to 36 dB by projecting point stimuli. An internal infrared camera allows precise stimulus projection through live retinal tracking based on the vessel image and live correction of the stimulus position. We used a 4–2 threshold strategy and, a testing grid with 6 degrees spacing, the Goldman stimulus size III, and a background luminance of 4 asb. The grid was composed of 37 points, with one central foveal point and 3 rings of 12 points each, with radii of 1 degree, 2 degrees, and 3 degrees, respectively. We chose this testing grid to take into account the lateral displacement of the retinal ganglion cells (RGCs) in the macula, as described by Drasdo et al. and Drasdo and Fowler.^24^^,^^25^ The maximum lateral displacement of RGC is 0.5 to 0.6 mm between 0.5 and 1.5-mm radius from the fovea, which corresponds to the tested visual field of 0.780 to 1.44-mm radius around the foveal pit on microperimetry.^26^^,^^27^ In addition, to analyze the relationship between the regional retinal sensitivity and the capillary density in each quadrant, the grid with 6 degrees spacing was used to better fit the parafoveal ring (0.5–1.5-mm radius) of the 3 × 3 mm parafoveal en face OCT-A images.^24^^,^^28^ In detail, the mean regional retinal sensitivity was manually calculated in each retinal quadrant (nasal, temporal, superior, and inferior) and correlated to the anatomic parameters provided by AngioVue software in the corresponding quadrant.

Studied variables were P1, P2, bivariate contour ellipse area (BCEA)@63%, and BCEA@95%. P1 and P2 corresponded to the percentage of fixation points falling inside a circle of 1 degree and 2 degrees radius, respectively, centered on the fovea. BCEA@63% and BCEA@95% corresponded to the area and orientation of an ellipse encompassing 63% and 95% of the fixation points, respectively.

No patient had ever undergone microperimetry. For this reason, statistical analyses were performed on all the eyes and also separately on the first and second eyes, to take into account the effects of the learning curve.

### OCT-A Imaging

All eyes were imaged using OCT-A (Angiovue, RTVue XR, Optovue, Inc.). OCT-A findings from a single visit were analyzed. A 3 mm × 3 mm macular cube centered on the fovea, composed of 320 horizontal B-scans separated by 9 µm and containing 320 A-scans, was acquired. Images were analyzed using the AngioVue OCT-A system available on the RTVue XR Avanti device. We used the terminology proposed by Campbell et al.^29^ to name the different retinal vascular plexuses. The preset parameters of the software were used to automatically segment the SCP and the DCC, whereas the deep capillary plexus (DCP) and the intermediate capillary plexus (ICP) were segmented manually. The SCP was comprised between the inner limiting membrane (ILM) and 9 µm above the junction between the inner plexiform layer and the inner nuclear layer (IPL-INL), whereas the DCC was comprised between 9 µm above the IPL-INL junction and 9 µm below the outer plexiform layer and outer nuclear layer (OPL-ONL) junction. The accuracy of the automatic segmentation was verified visually by scrolling the 320 B-scans. Then, we segmented manually the DCC into ICP and DCP by adjusting the segmentation boundaries. The ICP boundaries were set between 9 µm above the IPL-INL junction and 6 µm below the INL-OPL junction, thus including parts of the IPL and OPL and all the INL to record the ICP projection that is located in the IPL.^30^ The DCP boundaries were set between 6 µm below the INL-OPL junction and 9 µm below the OPL-ONL junction, thus including the OPL.^31^

Angioflow density was calculated in the SCP and the DCC using AngioAnalytics software including the Projection Artifact Removal (version 2015.1.0.71; Optovue, Inc.,). Details of the density calculation are undisclosed, but AngioAnalytics calculates the percentage of the angioflow surface relative to the total surface of the angiogram. The foveal avascular zone (FAZ) area was automatically measured using the FAZ function on OCT-A software, and manually corrected in case of segmentation errors.

The retinal layer thickness was also assessed on the same 3 mm × 3 mm OCT-A acquisitions. OCT-A software automatically segments the retinal layers and provides retinal thickness values within slabs of interest, from the ILM to the Bruch's membrane. In this study, three of the automatically generated slabs were selected for the analysis: the full retinal thickness slab from the ILM to the retinal pigment epithelium (RPE); the ganglion cell complex (GCC) slab from the nerve fiber layer to the IPL; and the INL slab from the IPL to the OPL. The segmentation of the different layers was manually corrected in case of segmentation errors. The VD and retinal thickness values were recorded for the whole 3 mm × 3 mm area, and for the foveal and parafoveal areas (circles of 1 mm and 3 mm in diameter centered on the fovea) corresponding to the ETDRS chart inner ring. The parafoveal regions of the ETDRS grid (3 mm in diameter) were used for the regional analysis of the VD in the SCP and the DCC.

### Image Analysis

We performed both quantitative and qualitative analyses.

For the quantitative analysis of the retinal sensitivity, the mean sensitivity was reported in decibels (dB) for the parafoveal ring and for each macular quadrant (temporal, superior, nasal, and inferior) and was calculated by transforming the sensitivity into a linear scale of the nine tested points, averaging the sensitivity on a linear scale, then returning to a decibel scale using the following formula: sensitivity (dB) = 10*Log10 (linear sensitivity).

Univariate and multivariate analyses were performed to assess the correlation between the mean sensitivity and patients’ demographics (age, diabetes duration, type of diabetes, and HbA1c levels), VA, OCT findings (central macular thickness [CMT], GCC, and INL thickness), OCT-A findings (FAZ area, VD in the SCP, DCC, ICP, and DCP), and microperimetry findings (P1, P2, BCEA@63%, and BCEA@95%).

**Figure 1.**
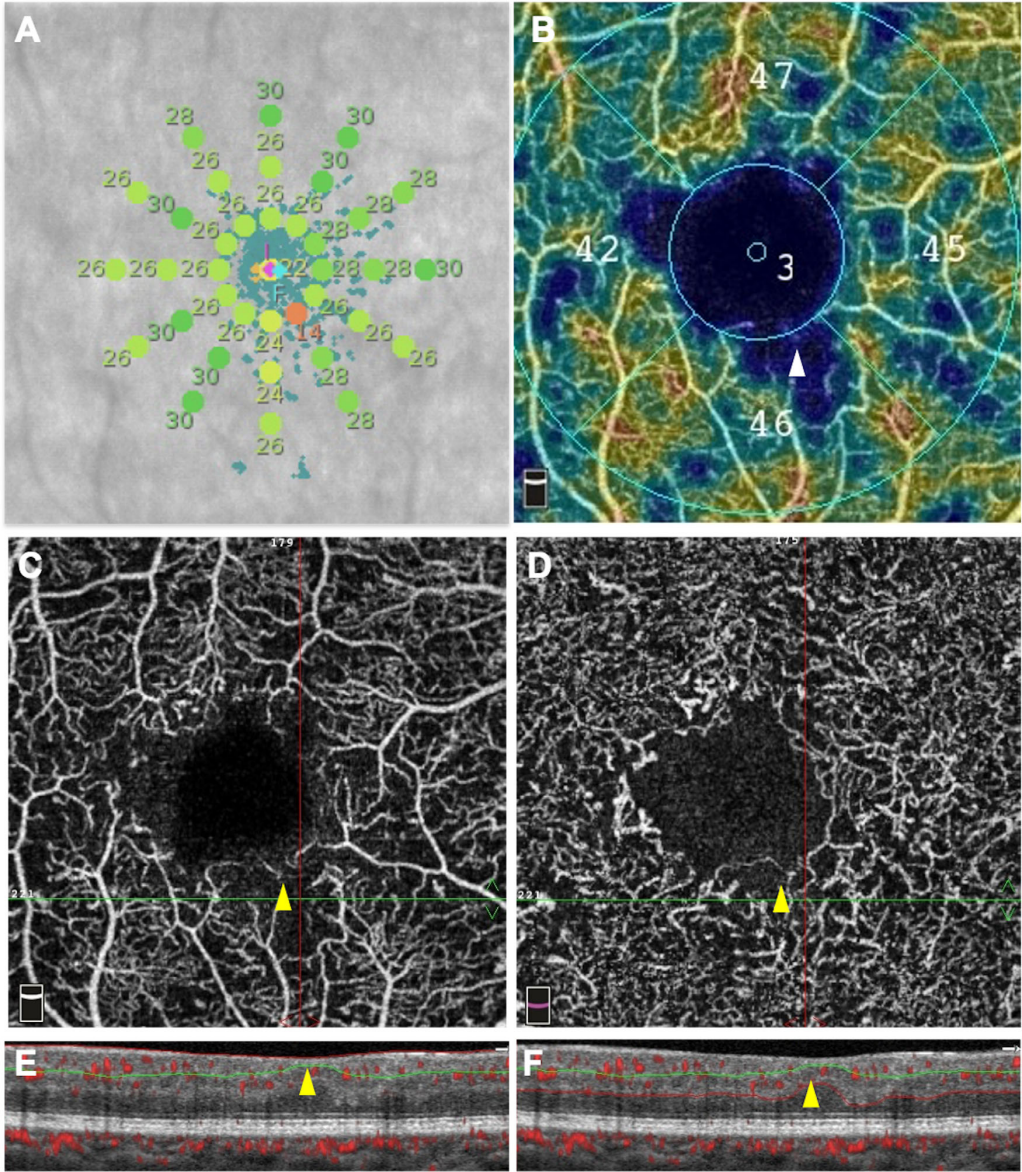
Microperimetric results (**A**) and OCT-angiography images (**B–F**) of an eye with moderate non-proliferative diabetic retinopathy. One point of decreased retinal sensitivity (14 dB) was detected by microperimetry **A** (*orange dot*) and co-located with an area of decreased capillary density on the superficial capillary plexus (SCP) color-coded density map **B** (*white arrowhead*), and on the SCP angiogram **C** (*yellow arrowhead*), as well as with an area of capillary loss in the deep capillary complex **D** (*yellow arrowhead*). The corresponding OCT B-scan with flow overlay **E** and **F** showed a focal ganglion cell complex thinning (*yellow arrowheads*) and a preserved outer retina.

To investigate the relationship between the regional retinal sensitivity and the VD and retinal thickness parameters, each OCT-A and microperimetry finding was assessed in the parafoveal ring to avoid a bias due to the FAZ area (Figs. 1A, 1B).

In the qualitative analysis, the capillary changes were assessed in both the SCP and the DCC in each area with a sensitivity <25 dB in each retinal quadrant.^32^ The capillary loss in the SCP was defined as delineated polygonal areas of capillary dropout present between the distal arterioles and venules, and in the DCC as a diffuse reduction in capillary density.^33^ The presence or the absence of a disorganization of the retinal inner layers (DRIL) and of external limiting membrane (ELM) and/or ellipsoid zone (EZ) disruptions were assessed in each area with a sensitivity <25 dB. All images were graded by two blinded retina specialists (authors A.M. and A.C.) and, in case of discrepancy between the graders, the images were reviewed for common agreement.

### Statistics

IBM SPSS statistics version 20 (IBM SPSS Statistics; IBM Corporation, Chicago, IL, USA) was used for the statistical analyses. Shapiro Wilk tests were used to assess the normal distribution of the data. To assess the association between the parafoveal sensitivity measured by microperimetry and the VD assessed by OCT-A in eyes with DR, univariate and multivariate analyses were performed. The association between the parafoveal sensitivity (and the sensitivity in each quadrant) measured by microperimetry and the explicative variables was assessed in a univariate analysis (a χ^2^ test or Fisher's exact test was used for categorial variables, and a *t*-test was used for quantitative variables). Spearman and Pearson correlation coefficients were used to assess nonlinear and linear correlations between the OCT thickness values, OCT-A findings, and microperimetry findings. A multivariate analysis was then performed using a multivariate linear regression model to assess the association between the parafoveal sensitivity measured by microperimetry and the variables. Different backward stepwise multivariate linear regressions, including the different variables, were used to identify the final model.^34^ All the variables were initially introduced in the model. The final model had the highest value of adjusted R². The age, gender, and HbA1c level were retained and forced in all multivariate analysis models. Data are presented as a mean ± SD for continuous variables and as a percentage for categorical variables. A *P* value <0.05 was considered significant.

## Results

Thirty-seven eyes of 21 patients were included in the study. The mean age was 51.3 ± 17.2 years (range = 22–82). Patients’ demographics and ocular characteristics are reported in Table 1.

**Table 1.** Patients’ Characteristics


Number of patients, *n*	21
Number of eyes, *n*	37
Gender: M/F, *n* (%)	9 (43)/12 (57)
Age (y), mean ± SD (range)	51.3 ± 17.2 (22–82)
Type of diabetes: type 1/ type 2, *n* (%)	11 (52)/10 (48)
Diabetes duration, (y) mean ± SD (range)	20.5 ± 15 (1–67)
HbA1c (mmol/mol), mean ± SD (range)	57.4 ± 9.8 (42–75)
BCVA (logMAR), mean ± SD (range)	0.01 ± 0.04 (−0.1–0.2)
Lens status: phakic, *n* (%)	31 (83)
**DR severity score**	
Absence of DR, *n* (%)	3 (8)
Mild NPDR, *n* (%)	11 (30)
Moderate NPDR, *n* (%)	6 (16)
Severe NPDR, *n* (%)	12 (32)
Proliferative DR inactivated by PRP, *n* (%)	5 (13)

BCVA, best-corrected visual acuity; SD, standard deviation; DR, diabetic retinopathy; NPDR, nonproliferative diabetic retinopathy; PRP, panretinal photocoagulation.

The mean parafoveal sensitivity was 28.1 ± 2.4 dB (range = 22–32). Regarding OCT-A findings, the mean parafoveal VD in the SCP was 43.2 ± 4.2% (range = 35–51) and the mean parafoveal VD in the DCC was 48.1 ± 3.3% (range = 40–56). All OCT-A and microperimetry quantitative parameters are shown in Table 2.

**Table 2.** OCT-Angiography (OCT-A) and Microperimetry Quantitative Parameters in the Whole Cohort (*n* = 37 Eyes of 21 Patients)

OCT-A Findings	
Central macular thickness (µm), mean ± SD (range)	263.2 ± 23.9 (208–317)
Mean parafoveal GCC thickness (µm), mean ± SD (range)	87.9 ± 13.5 (59–119)
Mean parafoveal INL thickness (µm), mean ± SD (range)	43.2 ± 9.9 (31–81)
Parafoveal VD in the SCP (%), mean ± SD (range)	43.2 ± 4.2 (35–51)
Parafoveal VD in the DCC (%), mean ± SD (range)	48.1 ± 3.3 (40–56)
Parafoveal VD in the ICP (%), mean ± SD (range)	41.0 ± 4.2 (31–49)
Parafoveal VD in the DCP (%), mean ± SD (range)	27.8 ± 4.4 (19–37)
FAZ area (mm^2^), mean ± SD (range)	0.31 ± 0.15 (0.12–0.75)
SSI, mean ± SD (range)	71.4 ± 6 (62–85)
**Microperimetry findings**	
Mean parafoveal sensitivity, mean ± SD (range)	28.1 ± 2.4 (22–32)
P1 (%), mean ± SD (range)	92.4 ± 7.6 (67–100)
P2 (%), mean ± SD (range)	98.8 ± 1.7 (93–100)
BCEA@63% (deg²), mean ± SD (range)	1.0 ± 0.8 (0–4)
BCEA@95% (deg²), mean ± SD (range)	3.1 ± 2.3 (1–11)

GCC, ganglion cell complex; INL, inner nuclear layer; SCP, superficial capillary plexus; DCC, deep capillary complex; ICP, intermediate capillary plexus; DCP, deep capillary plexus; VD, vessel density; BCEA, bivariate contour ellipse area; SD, standard deviation; FAZ, foveal avascular zone; SSI, signal strength intensity.

### Univariate Analysis

The univariate analyses are presented in Table 3. The mean retinal sensitivity correlated significantly and negatively with the age (R = −0.79, *P* < 0.001).

**Table 3.** Univariate Analyses of the Regional Retinal Sensitivity in the Parafoveal Ring With Patients’ Demographics, OCT and OCTA Parameters (*N* = 37)

Variables	Parafoveal Retinal Sensitivity R Coefficient	*P* Value
Age (y)	**−0.786**	**<0.001**
Diabetes duration (y)	−0.139	0.441
HbA1c (%)	−0.096	0.581
CMT (µm)	0.249	0.137
CCG thickness (µm)	**0.540**	**<0.001**
INL thickness (µm)	0.223	0.185
VD in the SCP (%)	**0.404**	**0.013**
VD in the DCC (%)	0.224	0.182
VD in the ICP (%)	0.218	0.194
VD in the DCP (%)	0.312	0.060
FAZ area (mm^2^)	**−0.367**	**0.027**
VA (logMar)	0.144	0.396

HbA1c, glycated hemoglobin A1c; CMT, central macular thickness; GCC, ganglion cell complex; INL, inner nuclear layer; VD, vessel density; SCP, superficial capillary plexus; DCC, deep capillary complex; ICP, intermediate capillary plexus; DCP, deep capillary plexus; FAZ, foveal avascular zone; VA, visual acuity.

Bold values indicate R coefficients with a significant *P* value <0.05.

The mean retinal sensitivity correlated significantly and positively with the VD in the SCP (R = 0.40, *P* = 0.013) and negatively with the FAZ area (R = −0.37, *P* = 0.027); although no significant correlation was found with the VD in the DCC, the ICP, and the DCP.

The mean regional retinal sensitivity correlated significantly and positively with the GCC thickness (R = 0.54, *P* < 0.001).

No significant correlation was found between the mean retinal sensitivity and the diabetes duration, VA, and HbA1c level.

The additional univariate analysis performed on each eye separately found the same correlation as for the whole cohort (Supplementary Table S1). No differences were found regarding the correlated parameters in these subgroups.

### Multivariate Linear Regression Model

All parameters were then tested in a multivariate linear regression model to confirm their correlation with the mean retinal sensitivity. In the multivariate linear regression model, the mean retinal sensitivity remained negatively correlated with the age (*P* = 0.04) and positively correlated with the VD in the SCP in the parafoveal ring (*P* = 0.01; Table 4). The mean sensitivity in the parafoveal ring also significantly and positively correlated with the INL thickness (*P* = 0.01).

**Table 4.** Multivariate Analysis of the Association Between the Parafoveal Retinal Sensitivity and the Covariables (*N* = 37)

Variables	Parafoveal Retinal Sensitivity Beta [95% CI]	*P* Value
Age (y)	−0.06 [−0.11; −0.001]	**0.04**
HbA1c (%)	−0.75 [−1.75; 0.25]	0.13
CMT (µm)	−0.02 [−0.05; 0.02]	0.4
CCG thickness (µm)	−0.01 [−0.09; 0.06]	0.72
INL thickness (µm)	0.11 [0.02; 0.19]	**0.01**
VD in the SCP (%)	0.29 [0.06; 0.52]	**0.01**
VD in the DCC (%)	−0.08 [−0.47; 0.3]	0.66
VD in the ICP (%)	0.002 [−0.24; 0.24]	0.99
VD in the DCP (%)	0.16 [−0.03; 0.36]	0.09

HbA1c, glycated hemoglobin A1c; 95% CI, 95% confidence interval; CMT, central macular thickness; GCC, ganglion cell complex; INL, inner nuclear layer; VD, vessel density; SCP, superficial capillary plexus; DCC, deep capillary complex; ICP, intermediate capillary plexus; DCP, deep capillary plexus.

Bold values indicate significant *P* value <0.05.

### Qualitative Analysis

**Figure 2.**
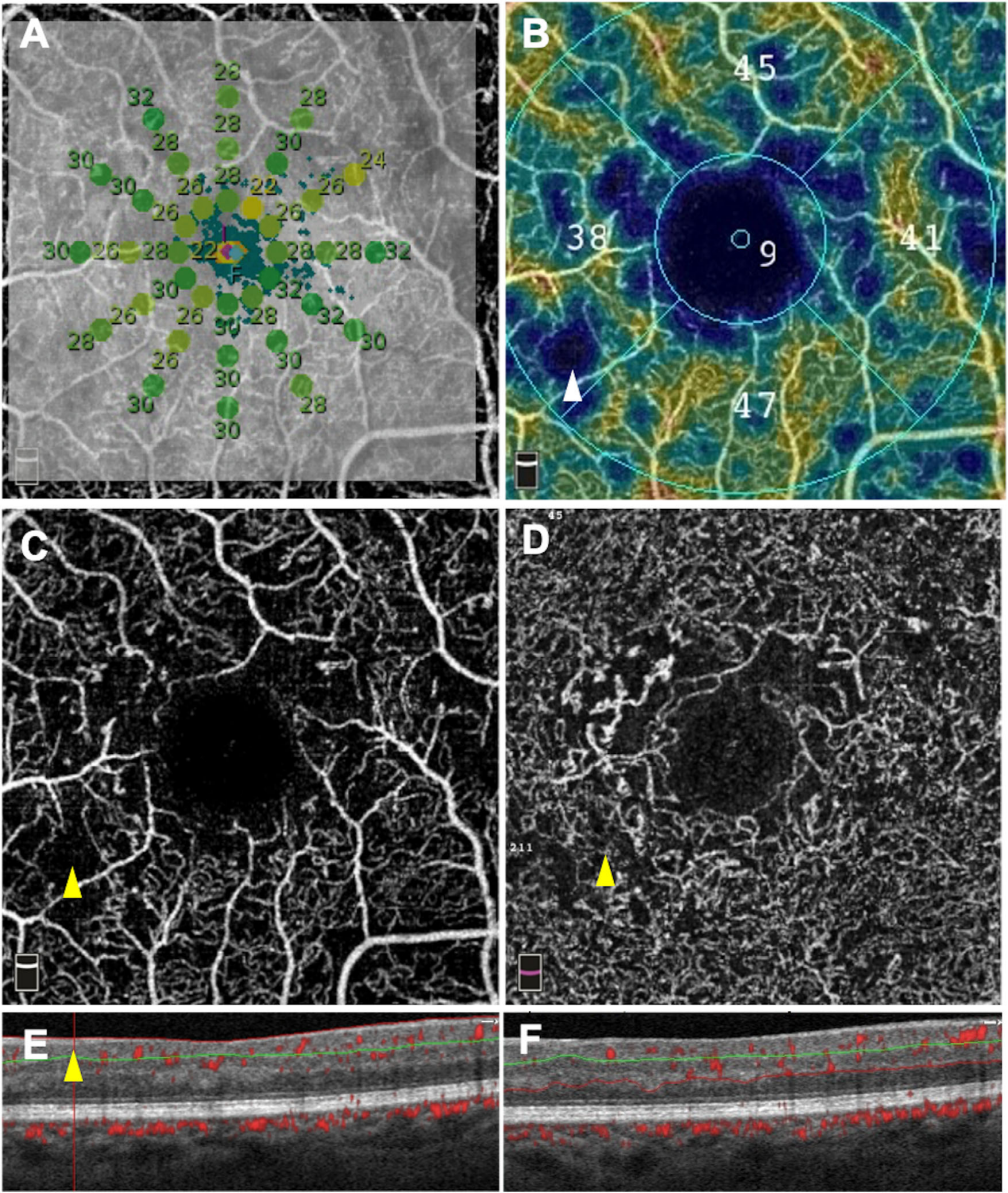
Microperimetric results (**A**) and OCT-angiography images (**B–F**) of an eye with severe nonproliferative diabetic retinopathy. The retinal sensitivity measured using microperimetry was greater than 25 dB in the inter-maculopapillary area **A** (*green dots*) whereas the capillary density in the superficial capillary plexus (SCP) was decreased on the color-coded density map **B** (*white arrowhead*). Accordingly, an area of capillary dropout was clearly visible on the SCP angiogram **C** (*yellow arrowhead*) in an area of almost normal retinal sensitivity (26 to 30 dB). An area of capillary dropout was also detected on the deep capillary complex angiogram in the corresponding area **D** (*yellow arrowhead*). The corresponding OCT B-scan with flow overlay **E** and **F** showed a focal ganglion cell complex thinning **E** (*yellow arrowheads*) and a preserved outer retina.

The capillary perfusion in each retinal area with a sensitivity <25 dB was assessed. The presence of at least 1 area with a sensitivity <25 dB was found in 45.9% (17/37) of eyes. All areas with a sensitivity <25 dB were co-located with areas of capillary dropout in the SCP slab and in the DCC slab (Figs. 1A–F). No DRIL and no ELM or EZ disruption were detected in the areas of retinal sensitivity <25 dB. Conversely, some areas of capillary dropout co-located with areas of normal retinal sensitivity (Fig. 2).

## Discussion

In this study, we aimed to assess the relationship between the macular sensitivity and the VD in eyes with DR but without DME and to investigate the capillary network in areas of decreased retinal sensitivity. The retinal sensitivity measured using microperimetry correlated with the corresponding local capillary areas on OCT-A and with the retinal layer thickness on the OCT B-scan in each quadrant and in the parafoveal ring. Our results showed that the mean retinal sensitivity correlated significantly and positively with the VD in the SCP and with the INL thickness in the parafoveal ring. Interestingly, the qualitative analysis of OCT-A images in each retinal quadrant showed that areas of capillary dropout did not correspond to absolute scotoma and could even have a normal retinal sensitivity. Conversely, all areas of decreased retinal sensitivity were always associated with capillary dropout.

In this series, only eyes without DME were included, and the mean retinal threshold was significantly decreased compared to a cohort of healthy controls previously published by Molina-Martin et al. despite a preserved VA,^35^ as already reported in DR.^19^ Regarding the anatomic parameters, the mean VD in the parafoveal ring was decreased in all plexuses compared to an age-matched healthy cohort from a previous study conducted by our team.^36^ The CMT and the INL thickness were normal because only eyes without DME were included, but the GCC thickness was reduced compared to our previously published age-matched healthy cohort.^36^ This finding was consistent with the neuroretinal degeneration that is known to occur in diabetic eyes.^37^

The correlation between the anatomic parameters and the visual function in DR is not fully understood. A weak correlation between the CMT and the VA has been reported,^38^ and the recent development of the OCT-A technology has allowed exploring new anatomic parameters. The relationship between the FAZ area or circumference and the VA has been studied by OCT-A, and only a weak correlation has been found.^9^^,^^10^^,^^39^ In a previous study focusing on a specific group of young patients with type 1 diabetes with severe rapidly progressive DR, we have shown that the VA was mainly conditioned by the VD in the DCC rather than by the FAZ area.^13^ In this previous study in type 1 diabetes eyes, a moderate loss of capillary perfusion in any retinal vascular plexus was compatible with a normal vision. Thus, it is likely that the VD decrease precedes the VA decrease. However, a significant VD decrease only in the DCP could be sufficient to induce a visual loss. This could be explained by the increased contribution of the DCP to photoreceptor metabolism in an ischemic environment. Similarly, in this study, a normal local sensitivity was observed in some areas of capillary dropout in the SCP and DCC. A local capillary loss is thus compatible with a preserved local retinal sensitivity, which could be due to a retinal metabolic autoregulation under certain conditions and to the remaining perfusion of the DCC, which was sufficient to maintain proper retinal function in these areas.

An impaired retinal sensitivity has been shown to precede the VA loss in DR. Thus, identifying correlations between the retinal sensitivity and the anatomic parameters could help to better understand the structural changes leading to the functional dysfunction in DR. A correlation between outer retina alterations and the VA in DR and DME eyes has been described.^40^^,^^41^ However, the point-by-point local relationship among the retinal sensitivity and capillary changes, inner retinal layer thinning, and EZ disruption, has not yet been assessed. Although DR is a generalized retinal disease, the loss of capillaries is usually not completely homogeneous in DR. It has been recently demonstrated that the correlation between the macular VD and the DR severity stage may not be present in eyes with predominant peripheral lesions.^39^ In this series, we performed a regional and local analysis to provide more precise information on the correlation between the retinal sensitivity and the anatomic parameters.

Our analysis of each retinal area with a low sensitivity (<25 dB) showed that the sensitivity could decrease in areas of capillary dropout despite a preserved outer retina. The preservation of the ELM and EZ has been associated with preserved VA and retinal sensitivity, and a significantly decreased retinal sensitivity has been shown in eyes with ELM and/or EZ disruptions.^42^ In this series, we observed areas of retinal sensitivity <25 dB in eyes with preserved ELM and EZ in the corresponding areas, while taking into account the displacement of the Henle fibers. The loss of capillaries and GCL-IPL thinning could thus be early events leading to the decrease in retinal sensitivity in eyes with DR, before the occurrence of any outer retinal alterations.

These focal areas of decreased retinal sensitivity have been recently described in one case of NPDR in areas of attenuated inner retinal layers on OCT but without loss of outer retinal structures.^43^ In patients with diabetes, Montesano et al. have described a significant correlation between the retinal sensitivity and the GCL-IPL thickness in diabetic eyes without DR. This suggests that the presence of inner retinal layer damage could deteriorate the visual function even before the appearance of any sign of vascular alterations. Our quantitative analysis found that the mean retinal sensitivity positively correlated with the VD in the SCP in the parafoveal ring. This correlation remained significant in the multivariate linear regression, including the age, which is a well-known confounding factor in retinal sensitivity assessment and could influence the VD.^44^^–^^47^ However, this correlation between the retinal sensitivity and the VD in the SCP was moderate in our series. Our qualitative analysis also showed that the sensitivity can be preserved in areas with capillary dropout. These results confirmed the complexity of the relationship between the anatomic parameters and the retinal sensitivity.

In our series, the retinal sensitivity correlated with the VD in the SCP but did not correlate with other OCT-A parameters (i.e. the VD in the DCC and the FAZ area). It is not clear why the retinal sensitivity correlated only with the VD in the SCP and not with the VD in the DCC. It could be assumed that the DCC is more resistant to chronic hyperglycemia-associated alterations because the VD decrease in the DCC compared to normal values was lower than the VD decrease in the SCP (compared to a previous age-matched healthy cohort^36^), and that some nonperfusion areas in the SCP could be co-located with a relatively preserved underlying DCC, as previously reported by our team.^33^ Another hypothesis could be that the capillary loss in the SCP is more easily delineated and measured as areas of capillary dropout compared to the capillary loss in the DCC that could be more generalized in the absence of dropout areas, due to its different anatomic architecture.^48^

Tsai et al. have also assessed the correlation between the retinal sensitivity and microvascular parameters in eyes with various degrees of DR severity.^21^ They have found that the retinal sensitivity correlated with an increased superficial FAZ area. In their series, the retinal sensitivity also correlated with a decreased VD in the DCC but not with the VD in the SCP, however, the Pearson correlation coefficients were relatively low for both. These variable results regarding the correlation between the retinal sensitivity and the VD could be related to the selection of patients because we included only a few patients without DR compared to the series by Tsai et al., to the difference in VA, and to the different devices used for retinal sensitivity and VD measurements.

This study has some limitations. First, it included a small number of eyes without follow-up and our results need to be confirmed in a larger series with a longitudinal analysis to explore the causal relationship between the structural changes and the decreased retinal sensitivity. In addition, we used a macular grid with a visual field span of 3 degrees from the fovea with a 36 dB testing range, which cannot be compared to the mean sensitivities reported in previous studies.^16^^,^^19^^,^^49^ The method does not take into account the intersubject variability, and a correction of the Drasdo model based on morphometric parameters that do not undergo pathological changes could help to provide more accurate calculations in the future.^28^ The choice of 3 × 3 mm scans to quantify VD limits the macular area scanned but it allows quantifying VD on higher quality scans because a wider scan size is known to be associated with a lower image resolution with the device used in this study.

Finally, eyes with DME or prior intravitreal treatments were not included in this study to avoid a bias in VD measurements due to the presence of intraretinal cystoid spaces or sequelae.

In conclusion, we showed that the parafoveolar sensitivity measured using microperimetry in eyes with DR without DME correlated positively with the VD in the SCP and with the INL thickness. Areas with a retinal sensitivity <25 dB were always associated with a capillary loss in both the SCP and the DCC without outer retinal alterations, however, some areas of capillary loss had a preserved retinal sensitivity. Longitudinal studies are needed to determine the strength of this correlation and to explore the exact sequence of macular structural changes leading to a decreased sensitivity and to the VA loss in DR.

## Supplementary Material

Supplement 1
