# Supplementary material for: Retinal Sensitivity Correlates With the Superficial Vessel Density and Inner Layer Thickness in Diabetic Retinopathy
Source: Invest Ophthalmol Vis Sci. 2021 Nov 30;62(14):28. doi: 10.1167/iovs.62.14.28 (PMC8648065; doi:10.1167/iovs.62.14.28)
Supplement: Supplement 1 [file iovs-62-14-28_s001.pdf]

	All eyes n= 37				1st eyes n= 20				2nd eyes n = 17			
	Temporal	Superior	Nasal	Inferior	Temporal	Superior	Nasal	Inferior	Temporal	Superior	Nasal	Inferior
Age	-0.623	-0.494	-0.557	-0.636	-0.638	-0.502	-0.549	-0.687	-0.606	-0.480	-0.593	-0.537
p value	0.000*	0.002*	0.000*	0.000*	0.002*	0.024*	0.012*	0.001*	0.010*	0.050*	0.012*	0.028*
Duration of diabetes	-0.205	-0.240	-0.045	0.096	-0.143	-0.053	-0.092	-0.217	0.484	0.382	0.377	0.287
p value	0.224	0.153	0.790	0.573	0.547	0.825	0.700	0.359	0.069	0.160	0.166	0.297
Full retinal Thickness £	0.404	0.115	0.466	0.580	0.430	0.418	0.403	0.612	0.420	0.035	0.431	0.581
p value	0.108	0.6595	0.060	0.0162*	0.058	0.066	0.078	0.004*	0.093	0.894	0.084	0.016*
GCC thickness	0.579	0.375	0.388	0.728	0.569	0.362	0.319	0.695	0.632	0.397	0.586	0.706
p value	0.000*	0.022*	0.018*	0.000*	0.009*	0.116	0.170	0.001*	0.008*	0.116	0.015*	0.002*
INL thickness £	-0.191	-0.134	0.138	0.019	-0.264	-0.376	-0.117	0.102	-0.191	-0.135	0.139	0.020
p value	0.460	0.605	0.593	0.941	0.261	0.102	0.622	0.668	0.461	0.605	0.594	0.942
SVC Vessel Density	0.390	0.396	0.330	0.507	0.349	0.302	0.207	0.240	0.476	0.641	0.535	0.601
p value	0.017*	0.015*	0.046*	0.001*	0.031*	0.025*	0.038*	0.031*	0.053*	0.006*	0.027*	0.012*
DVC Vessel Density	0.111	0.125	0.314	0.149	-0.031	0.012	0.250	0.019	0.306	0.435	0.318	0.146
p value	0.513	0.460	0.058	0.380	0.897	0.959	0.288	0.938	0.232	0.081	0.213	0.574
ICP Vessel Density	0.267	0.056	0.379	0.249	0.086	-0.089	0.338	0.151	0.459	0.253	0.313	0.167
p value	0.110	0.742	0.021*	0.137	0.719	0.710	0.145	0.525	0.064	0.327	0.222	0.523
DVP Vessel Density	0.270	0.205	0.263	0.268	0.280	0.340	0.246	0.156	0.306	0.165	0.331	0.284
p value	0.106	0.225	0.116	0.109	0.232	0.142	0.296	0.510	0.233	0.527	0.194	0.268
DR Severity £	0.217	0.115	0.305	0.244	-0.025	-0.018	0.203	0.200	0.112	-0.137	0.009	-0.030
p value	0.197	0.497	0.066	0.145	0.91	0.937	0.390	0.397	0.667	0.598	0.971	0.908

Supplementary table 1 : R coefficient for correlation univariate analyses of retinal sensitivity in each retinal quadrant with demographics. OCT and OCTA parameters. performed on each eye. (* = p <0.05)
